# Navigated Intraoperative 3D Ultrasound in Glioblastoma Surgery: Analysis of Imaging Features and Impact on Extent of Resection

**DOI:** 10.3389/fnins.2022.883584

**Published:** 2022-05-09

**Authors:** Benjamin Saß, Darko Zivkovic, Mirza Pojskic, Christopher Nimsky, Miriam H. A. Bopp

**Affiliations:** ^1^Department of Neurosurgery, University of Marburg, Marburg, Germany; ^2^Center for Mind, Brain and Behavior (CMBB), Marburg, Germany

**Keywords:** glioblastoma, intraoperative ultrasound, intraoperative imaging, brain shift, neuronavigation, extent of resection

## Abstract

**Background:**

Neuronavigation is routinely used in glioblastoma surgery, but its accuracy decreases during the operative procedure due to brain shift, which can be addressed utilizing intraoperative imaging. Intraoperative ultrasound (iUS) is widely available, offers excellent live imaging, and can be fully integrated into modern navigational systems. Here, we analyze the imaging features of navigated i3D US and its impact on the extent of resection (EOR) in glioblastoma surgery.

**Methods:**

Datasets of 31 glioblastoma resection procedures were evaluated. Patient registration was established using intraoperative computed tomography (iCT). Pre-operative MRI (pre-MRI) and pre-resectional ultrasound (pre-US) datasets were compared regarding segmented tumor volume, spatial overlap (Dice coefficient), the Euclidean distance of the geometric center of gravity (CoG), and the Hausdorff distance. Post-resectional ultrasound (post-US) and post-operative MRI (post-MRI) tumor volumes were analyzed and categorized into subtotal resection (STR) or gross total resection (GTR) cases.

**Results:**

The mean patient age was 59.3 ± 11.9 years. There was no significant difference in pre-resectional segmented tumor volumes (pre-MRI: 24.2 ± 22.3 cm^3^; pre-US: 24.0 ± 21.8 cm^3^). The Dice coefficient was 0.71 ± 0.21, the Euclidean distance of the CoG was 3.9 ± 3.0 mm, and the Hausdorff distance was 12.2 ± 6.9 mm. A total of 18 cases were categorized as GTR, 10 cases were concordantly classified as STR on MRI and ultrasound, and 3 cases had to be excluded from post-resectional analysis. In four cases, i3D US triggered further resection.

**Conclusion:**

Navigated i3D US is reliably adjunct in a multimodal navigational setup for glioblastoma resection. Tumor segmentations revealed similar results in i3D US and MRI, demonstrating the capability of i3D US to delineate tumor boundaries. Additionally, i3D US has a positive influence on the EOR, allows live imaging, and depicts brain shift.

## Introduction

With a total of 49.1%, glioblastomas are the most common malignant primary brain tumors in the United States, accounting for 14.3% of all primary brain tumors, while showing the lowest median survival time of 8 months (Ostrom et al., 2021), in spite of advances in therapy, including surgery, radiation, chemotherapy (Stupp et al., 2005, 2009), and alternating electric fields [tumor treating fields (TTFs)] (Burri et al., 2018). The extent of resection (EOR) and residual tumor volume are known to be significant factors influencing the patient overall survival time as demonstrated by several studies supporting the superiority of gross total resection (GTR) over subtotal resection (STR) or biopsy (Grabowski et al., 2014; Brown et al., 2016). In fact, compared to STR, GTR increases the likelihood of 1-year survival about 61% and the likelihood of 2-year survival about 19% (Brown et al., 2016). Furthermore, resection of fluid-attenuated inversion recovery (FLAIR) abnormalities beyond the boundaries of T1 contrast-enhancing tumor regions was found to additionally prolong median survival (Li et al., 2016). In contrast, extended resection increases the risk of neurological impairment (Stummer et al., 2011), which in turn decreases the likelihood of consequent adjuvant therapy (Gulati et al., 2011). Modern neurosurgical intraoperative setups include neuronavigational systems, multimodal magnetic resonance imaging data, if available intraoperative MRI (iMRI), intraoperative stimulation mapping, and 5-aminolevulinic acid supported techniques to minimize the risk of neurological deterioration while maximizing the EOR (Stummer et al., 2006; De Witt Hamer et al., 2012; Wen et al., 2020). Because neuronavigational systems are mainly based on pre-operative imaging, they harbor the risk of an increasing inaccuracy during the operative course due to brain shift, a phenomenon, which can be attributed to influences of gravity, brain retraction and swelling, tissue removal, and loss of cerebrospinal fluid (Roberts et al., 1998; Nimsky et al., 2000). Attempts to quantify intraoperative brain shift were made as early as by Kelly et al. (1986) who developed an approach using radiographic imaging for the detection of movement of intracranially inserted reference balls. Others tested systems based on the analysis of video images and microscope tracking (Roberts et al., 1998), optical scanning (Audette et al., 1999), or simply a navigated pointer (Dorward et al., 1998; Hill et al., 1998). Intraprocedural displacement of cortical and deeper located structures is not correlated, demonstrating the complexity of brain deformation (Hastreiter et al., 2004). Moreover, the phenomenon occurs throughout the surgical procedure and, consequently, only repetitive intraoperative imaging can provide the basis for accurate image guidance (Nabavi et al., 2001).

The two major modalities in intraprocedural imaging are iMRI and intraoperative ultrasound (iUS) (Wirtz et al., 1997; Nimsky et al., 2000, 2001; Nabavi et al., 2001). Despite the indisputable advantage of excellent imaging quality, iMRI has its shortcomings, such as limited availability, high costs, the need for a special training of the staff, structural requirements, and high time consumption (Nimsky et al., 2004; Reinertsen et al., 2014). In contrast, today iUS is widely available, cost effective, straightforward in use, and even multiple image acquisitions can be easily integrated into the surgical procedure (Sastry et al., 2017). Although iUS was already introduced (Chandler et al., 1982) and routinely used by some neurosurgeons in the 1980s (Knake et al., 1982), it was not widely accepted until neuronavigational systems and high imaging quality iUS coalesced (Gronningsaeter et al., 2000; Ohue et al., 2010). The consistent further development of navigated intraoperative 3D ultrasound (i3D US) (Tronnier et al., 2001; Unsgaard et al., 2002) allows comfortable sequentially updating of 3D imaging maps during surgery (Unsgaard et al., 2002) and thus may be a supportive measure for estimating brain shift and the EOR in glioma surgery during the procedure (Munkvold et al., 2018).

In our institution, i3D US has become an integral part of the surgical routine for intracranial space-occupying lesions. In cases of brain metastasis, we have shown that i3D US clearly delineates tumor boundaries, allows live imaging updates, and identifies brain deformation reliably (Saß et al., 2020). In contrast to metastases, gliomas feature less well-defined boundaries and, apparently, depicting these in iUS is more difficult. Thus, we initially conceptualized a proof-of-concept study, which demonstrated the use of intraoperative Doppler for the visualization of the shift of vascular structures as an instrument for brain shift estimation in glioma surgery. However, this approach did not provide direct information on the tumor itself or the EOR (Sass et al., 2021). Consequently, here we present our analysis of 31 prospectively obtained intraoperative computed tomography (iCT) registration-based navigated i3D US datasets in glioblastoma patients. By addressing tumor volume, shape, and shifting when compared to pre-operative MRI (pre-MRI), as well as the subjective impression of imaging quality, and the impact of i3D US on EOR, we describe the benefits as well as the drawbacks and pitfalls of navigated i3D US in a modern multimodal setup.

## Materials and Methods

This prospective study encompasses all surgical cases of primary or recurrent glioblastoma in 2020 operated with iCT-based registration for a total of 31 cases in 29 patients (case nos. 9 and 10, and case nos. 5 and 26 were the same patients, respectively). In all cases, surgery was recommended by a neuro-oncological tumor board, and all patients provided written informed consent. Ethics approval for archiving clinical and technical data applying intraoperative imaging and navigation was obtained (study no. 99/18).

Within a few days before surgery, all patients underwent a pre-operative stocktickerMRI, encompassing at least native and contrast-enhanced T1-weighted, T2-weighted, and FLAIR sequences. Pre-operative imaging was transferred to the navigational system (Brainlab, Munich, Germany), which consisted of a ceiling-mounted double monitor (Curve, Brainlab, Munich, Germany), two wall-embedded screens (Buzz, Brainlab, Munich, Germany), the navigational camera, server, and software (Elements, Brainlab, Munich, Germany). Within the software, the computer-assisted outlining tool called smart brush was used to segment the tumor masses in the T1-weighted contrast-enhanced stocktickerMRI, or in cases of little enhancement, in T2-weighted or FLAIR sequences. In this application, the user marks roughly the tumor in one slice and the software automatically delineates the tumor boundaries. To create a 3D object, the same procedure is repeated in a perpendicular plane. Then, the 3D object is reviewed by the surgeon and if necessary edited to achieve the best possible segmentation result. Additionally, eloquent regions at risk were outlined either using the smart brush application or the automatic cranial segmentation application, which features automated, knowledge-based anatomical contouring.

The surgical procedures were conducted under general anesthesia. All patients received 40 mg of dexamethasone after narcosis induction and perioperative antibiotic prophylaxis. The patients then were positioned on an operating room (OR) table and the head was fixed to the table using a radiolucent DORO^®^ Skull with metal pins. Whenever possible, the pins were positioned in a manner that the surgical field could be scanned between the pins, reducing metal artifacts. A reference array with four reflective markers was attached to the head clamp. The registration scan was either performed before skin incision or after craniotomy. If performed before skin incision, three fiducial markers were placed within the anticipated scanning region. The navigation camera was then aligned to the reference array and reflective markers on the iCT. Next, the OR table was rotated 90° to the mobile 32-slice CT-scanner (AIRO, Brainlab, Munich) and a low-dose registration scan (0.042 mSv) of 62 mm scan length was performed, while the camera detected the reference array and reflective markers on the scanner. Once the scan was completed, the table was turned back, and the camera was adjusted to detect the reference array. The iCT datasets were automatically transferred to the navigational system and fused to the pre-operative imaging data for automatic patient registration. The three fiducial markers were used to determine the registration accuracy. To accomplish this, the tip of a navigational pointer was placed in the divot of each fiducial marker, cross-checked, and saved to the navigational system, which allowed the calculation of the target registration error (TRE) as the Euclidean offset of the pointer tip subsequently. The navigational system was then ready to use, and the cranial approach could be planned. Details of the iCT-based registration procedure were published before (Carl et al., 2018).

After team time-out, skin disinfection, and sterile draping the skin was incised and the cranial bone exposed. Immediately before the craniotomy was performed, the patient received 125 ml of 15% mannitol. In cases where the registration scan was performed after craniotomy, the approach was planned based on anatomical landmarks. In these cases, the scanning procedure itself was similar to the one mentioned above, but under sterile conditions, and an additional scout scan was performed to define the 62 mm scan region. This modified procedure did not allow the aforementioned TRE calculation, and the registration accuracy had to be reviewed comparing landmarks at the patient’s head (e.g., at the edge of the craniotomy or superficial cortical vessels) to the displayed navigational data.

Reasoned by our experience that the imaging quality is the highest before dural opening, and to allow as less potentially harmful direct contact of the ultrasound probe and the brain surface as possible, the first navigated i3D US datasets were acquired directly after bone removal but before dura incision utilizing the bk5000 system (bk medical, Herlev, Denmark) connected to the navigational system. The scan was performed with an immersible, sterilizable high-resolution small footprint transducer (N13C5, bk medical, Herlev, Denmark), which has a 29 mm × 10 mm convex scanning surface and a scanning range from 5 to 13 MHz. Saline was administered as a coupling fluid, then the pre-calibrated transducer, which was equipped with a reference array, was smoothly moved over the dura generating 2D slices, which were automatically transferred to the navigational system. Here, the 0.3 mm 2D slices were automatically processed to co-registered 3D datasets, which could be further displayed as an overlay with the pre-operative imaging, side-by-side, or as a stand-alone and could be reformatted into any orientation.

**FIGURE 1:**
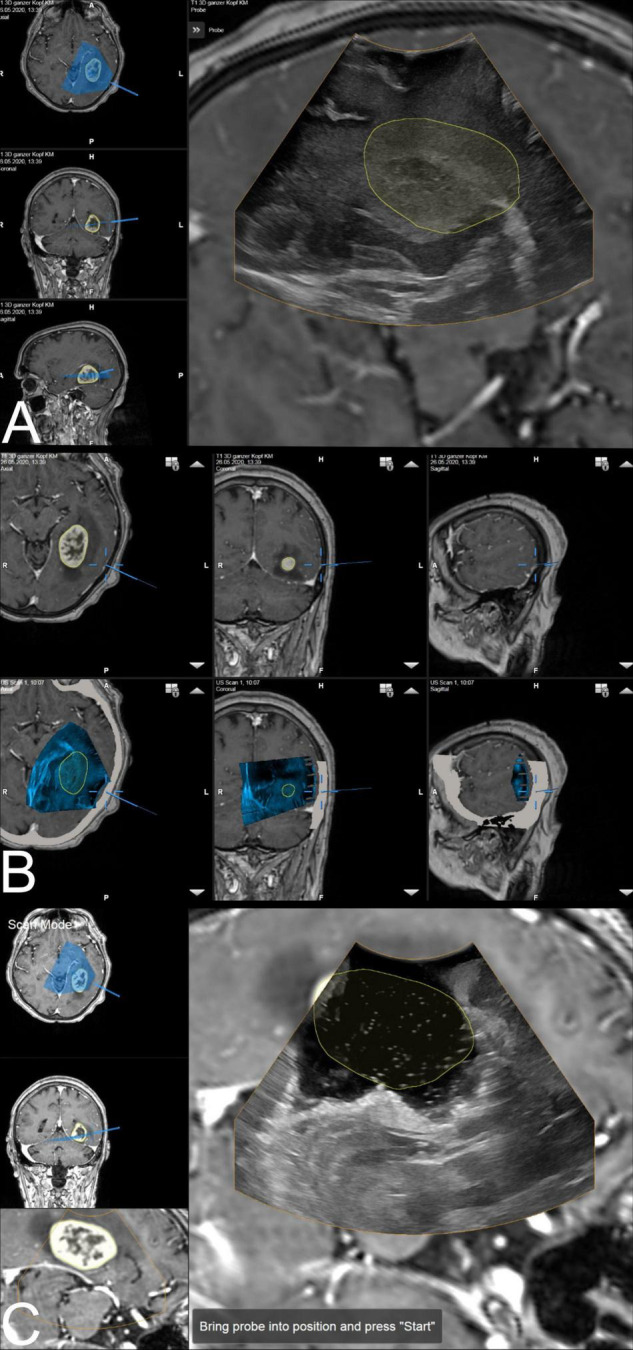
The ultrasound application: intraoperative display of i3D US over MRI data. **(A)** The first intraoperative ultrasound before dural opening is displayed as an overlay. The yellow line shows the tumor borders segmented on pre-MRI, which are not correspondent with i3D US, indicating brain deformation. Corresponding axial, coronal, and sagittal view of pre-MRI data is shown on the left side, including a visualization of localization and orientation of the recent i3D US. **(B)** After i3D US acquisition, the ultrasound application automatically displays pre-operative imaging (upper row: pre-MRI) and intraoperative imaging (lower row: i3D US as an overlay in pre-MRI) simultaneously in an axial, coronal, and sagittal slice depending on the position of the navigated transducer (cursor). Besides this, the arrows on the right side of each view allow for scrolling through the co-registered i3D US and pre-MRI sets to further explore the data. **(C)** The second navigated intraoperative ultrasound after resection (post-US) is displayed as an overlay on the pre-MRI. Analogous to **(A)**, the tumor outlines based on the pre-MRI data are visualized in yellow demonstrating the tumor boundaries to be completely within the resection cavity. For comparison of live ultrasound images and pre-MRI data, the scan mode view is chosen. In this case, the left-hand side of the image shows the axial and coronal view, as well as the inline view of the pre-MRI dataset, on which the ultrasound data is superimposed, without the overlay.

After tumor removal, a saline depot was filled in the resection cavity as coupling fluid and a second 3D scan was performed and evaluated. In some cases, when residual tumor volume was detected and resection was thereof extended, another scan was carried out. Additional scans were acquired at any time during surgery if deemed necessary by the surgeon. Figure 1 illustrates the above-mentioned steps of i3D US application.

During the surgical procedure, the overlay or side-by-side view of pre-MRI and i3D US datasets was helpful in the rough estimation of brain shift. For more detailed analysis, the lesions or residual tumor volumes were segmented in the navigational software using the smart brush application. Pre-operative and intraoperative data were exported to MeVisLab (MeVis Medical Solutions AG, Bremen, Germany) for further processing, analogous to the procedure we described for brain metastases (Saß et al., 2020).

An estimation of the displacement of the segmented object was investigated by calculation of the Euclidean offset of the geometric centers of gravity (CoG), which were calculated by summing up the coordinates of voxels and dividing them by the number of voxels. The following formula was used to calculate the CoG, where *A*_*US*_ and *B*_*MR*_ are sets of voxels of the segmented objects in US and MRI datasets, and *a* and *b* are the coordinates of the voxels:


$$C\,o\,G_{U\,S}=\frac{1}{\left|A_{U\,S}\right|}\times \sum_{a\in A_{U\,S}}a\,a\,n\,d\,C\,o\,G_{M\,R}=\frac{1}{\left|B_{M\,R}\right|}\times \sum_{b\in B_{M\,R}}b$$

The geometric CoG is a robust measure under rotation, scaling, and skewing, not susceptible to random noise (Flusser and Suk, 1994), and has thus been used for similar displacement measurements before (Paul et al., 2015).

As a similarity measure, the spatial overlap of the segmented object was determined using the Dice coefficient (C_*DSC*_) (Dice, 1945; Zou et al., 2004; Nitsch et al., 2019):


$$C_{D\,s\,c}=\frac{2\,\left|A_{U\,S}\,⋂B_{M\,R}\right|}{\left|A_{U\,S}\right|+\left|B_{M\,R}\right|}$$

The C_*DSC*_ is a value between 0 and 1, where 0 indicates no overlap and 1 indicates an exact match (Nitsch et al., 2019).

The resemblance of objects was evaluated with the Hausdorff distance, which measures the distance of each voxel of one segmentation that is the farthest from any point of the other segmentation and *vice versa* (Huttenlocher et al., 1993) and is defined as


(1) $$H\,\left(A_{U\,S},B_{M\,R}\right)=\max\left\{d_{m\,a\,x}\,\left(A_{U\,S},B_{M\,R}\right),d_{m\,a\,x}\,\left(B_{M\,R},A_{U\,S}\right)\right\}$$

$$\text{with}\,d_{m\,a\,x}=\max_{a\in A_{U\,S}}\min_{b\in B_{M\,R}}||a-b||$$

(2) (Huttenlocheret al.,1993;Nitschet al.,2019).

The Hausdorff distance, which is measured in millimeter, is smaller the more the segmentations resemble each other (Nitsch et al., 2019).

Data were statistically analyzed with GraphPad Prism 8.4.3 (GraphPad Software, San Diego, CA, United States) for Mac OS. The D’Agostino-Pearson test was used for testing for normal distribution. The Wilcoxon matched-pairs signed-rank test was used for not normally distributed, paired data analysis.

A *p*-value of < 0.05 was considered statistically significant.

## Results

A total of 31 cases in 29 patients (12 females, 17 males) were included in this study. The mean ± standard deviation (SD) age at time of surgery was 59.3 ± 11.9 years, ranging from 29.4 to 77.9 years. The most common glioblastoma location was the frontal lobe (12; right:left = 9:3) followed by temporal (9; right:left = 6:3), temporo-parieto-occipital (6; right:left = 5:1), parietal (2; right:left = 1:1), occipital (1: right), and cerebellar (1; right). Histological workup revealed isocitrate dehydrogenase (IDH) wild-type-glioblastoma WHO grade IV in 29 cases, of which case no. 20 featured a giant cell component, case no. 26 was described as infiltration zone of a known glioblastoma, and histology in case no. 30 was mainly necrosis. Case nos. 2 and 15 were IDH-mutant glioblastoma grade IV. For more details on patient characteristics, refer to Table 1.

**TABLE 1:** Patient characteristics.

Case	Age [years]	Sex	Localization/Site	Neuropathological diagnosis/IDH status	Primary/Recurrent
1	58.16	f	Temporal/r	GBM grade IV/IDH wt	P
2	29.38	m	Frontal/l	GBM grade IV/IDH mut	P
3	61.63	f	Frontal/l	GBM grade IV/IDH wt	P
4	47.50	m	Parietal/r	GBM grade IV/IDH wt	R
5	44.52	m	Frontal/r	GBM grade IV/IDH wt	R
6	62.39	m	Temporal/r	GBM grade IV/IDH wt	P
7	67.74	f	Frontal/r	GBM grade IV/IDH wt	P
8	52.43	m	Tpo/r	GBM grade IV/IDH wt	R
9	63.22	f	Frontal/r	GBM grade IV/IDH wt	R
10	63.23	f	Frontal/r	GBM grade IV/IDH wt	R
11	68.98	m	Cerebellar/r	GBM grade IV/IDH wt	R
12	57.88	f	Temporal/r	GBM grade IV/IDH wt	P
13	77.91	f	Temporal/l	GBM grade IV/IDH wt	P
14	73.96	f	Tpo/r	GBM grade IV/IDH wt	R
15	40.76	m	Temporal/r	GBM grade IV/IDH mut	R
16	47.63	m	Parietal/l	GBM grade IV/IDH wt	P
17	68.91	m	Frontal/l	GBM grade IV/IDH wt	P
18	76.62	f	Frontal/r	GBM grade IV/IDH wt	P
19	68.99	m	Frontal/r	GBM grade IV/IDH wt	R
20	54.53	m	Temporal/l	GBM grade IV/IDH wt (with a giant cell component)	P
21	73.09	f	Temporal/r	GBM grade IV/IDH wt	R
22	55.38	m	Tpo/r	GBM grade IV/IDH wt	R
23	61.69	m	Frontal/r	GBM grade IV/IDH wt	P
24	64.48	m	Occipital/r	GBM grade IV/IDH wt	P
25	66.82	m	Temporal/l	GBM grade IV/IDH wt	P
26	45.17	m	Frontal/r	GBM grade IV/IDH wt (infiltration zone)	R
27	75.90	m	Tpo/l	GBM grade IV/IDH wt	P
28	55.58	f	Temporal/r	GBM grade IV/IDH wt	P
29	64.77	f	Tpo/r	Necrosis, GBM grade IV/IDH wt	R
30	43.25	m	Tpo/r	GBM grade IV/IDH wt	R
31	47.25	f	Frontal/r	GBM grade IV/IDH wt	P

*f, female; GBM, glioblastoma; IDH, isocitrate dehydrogenase; m, male; mut, mutant; P, primary; R, recurrent; tpo, temporo-parieto-occipital; wt, wildtype.*

Due to low imaging quality in pre-resectional i3D US (pre-US), case no. 15 had to be excluded from pre-resectional analysis. In 14 cases (registration scan performed prior to skin incision), the mean TRE was calculated, resulting in a mean ± SD of 0.91 ± 0.53 mm, which is comparable to our previous published data (Carl et al., 2018). The imaging quality of i3D US was subjectively judged as excellent in 17 cases (55%), good in 8 cases (26%), sufficient in 5 cases (16%), and poor in 1 case (3%), depending on the amount of artifacts and the quality of established of contact between the probe’s surface and the dural layer.

**FIGURE 2:**
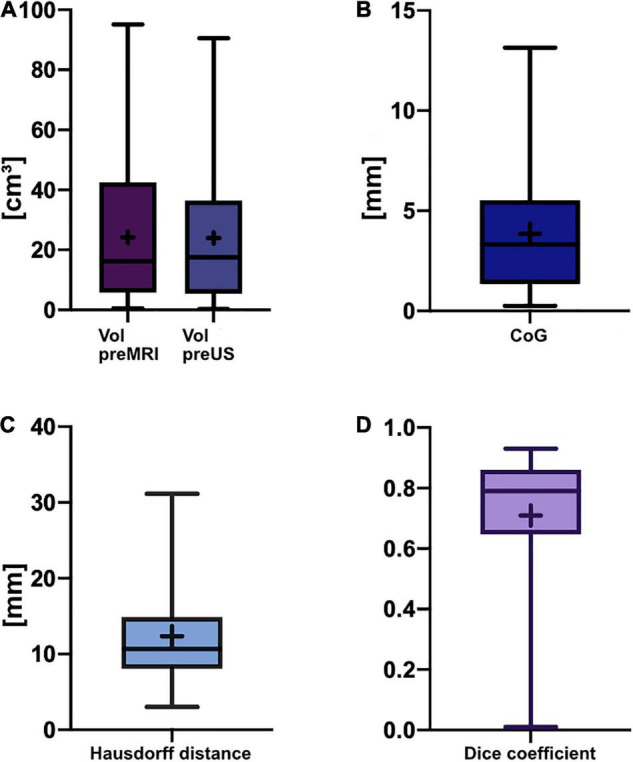
Box-and-whisker plots of pre-resectional data. The lines indicate the medians, boxes extend from the 25th to 75th percentile, the whiskers encompass the range; + indicates the mean. **(A)** Tumor volumes: Vol pre-MRI 24.2 ± 22.3 cm^3^ (mean ± SD) and median 16.3 cm^3^, Vol pre-US 24.0 ± 21.8 cm^3^ (mean ± SD) and median 17.6 cm^3^; no significant difference (*p* = 0.8752, two-tailed Wilcoxon-matched pairs test). **(B)** Euclidean distance of the center of gravity: 3.9 ± 3.0 mm (mean ± SD), median of 3.3 mm. **(C)** Hausdorff distance: 12.3 ± 6.9 mm (mean ± SD), median of 10.7 mm. **(D)** Dice coefficient: 0.71 ± 0.21 (mean ± SD), median of 0.79 (unitless).

The mean pre-operative tumor volume segmented in MRI (Vol pre-MRI) was 24.2 ± 22.3 cm^3^ (mean ± SD) with a median of 16.3 cm^3^ compared to 24.0 ± 21.8 cm^3^ (mean ± SD) with a median of 17.6 cm^3^ when segmented in the pre-resectional ultrasound datasets (Vol pre-US;, refer to Figure 2A). Both groups were not normally distributed (D’Agostino-Pearson test), and the two-tailed Wilcoxon matched-pairs test demonstrated no significant difference (*p* = 0.8752) with a median of differences of −0.005 cm^3^, whereas the median of the magnitude of the differences, which was calculated to overcome the issue of balancing negative and positive values, was 0.95 cm^3^.

Pre-resectionally, the Euclidean distance of the CoG was 3.9 ± 3.0 mm (mean ± SD) with a median of 3.3 mm, whereas the Hausdorff distance was 12.3 ± 6.9 mm (mean ± SD) with a median of 10.7 mm (Figures 2B,C). The mean Dice coefficient C_*DSC*_ was 0.71 ± 0.21 (mean ± SD) with a median of 0.79 (Figure 2D). The lowest C_*DSC*_ was found in case no. 25, where the tumor volume was extraordinarily small (Vol pre-MRI: 0.5 cm^3^). However, C_*DSC*_ did not correlate with tumor size in pre-operative MRI (*r* = 0.34, *p* = 0.0675, non-parametric Spearman correlation) or pre-resectional i3D US (*r* = 0.33, *p* = 0.0738, non-parametric Spearman correlation).

Three cases had to be excluded from statistical analysis of post-resectional volumes for different reasons (see below). The post-operative tumor volume segmented in MRI (Vol post-MRI) was 1.3 ± 3.5 cm^3^ (mean ± SD), which is similar to the post-resectional tumor volume segmented in the last ultrasound (Vol post-US), which was 1.2 ± 3.3 cm^3^. In 18 of 28 cases, the last acquired i3D US tumor volume was 0.0 cm^3^ (complying with GTR), but in 10 cases i3D US revealed remaining tumor volume, but no further resection was indicated, thus, those cases were classified as STR. Vol post-US in these cases was 3.5 ± 4.9 cm^3^ (mean ± SD) with a median of 2.1 cm^3^ and Vol post-MRI was 3.5 ± 5.2 cm^3^ (mean ± SD) with a median 2.4 cm^3^, and did not significantly differ (two-tailed Wilcoxon matched-pairs test, *p* = 0.8457). Additionally, in four cases where total resection was planned (GTR-cases), i3D US demonstrated a significant remaining tumor, which led to extension of tumor resection.

The excluded cases were case nos. 7 and 8, which had corrupted post-resectional i3D US datasets and case no. 9, which was excluded, because it was the same patient as case no. 10, who underwent surgery two times within 5 days. In this case, tumor progression with a volume of 3.0 cm^3^ was identified in the post-operative MRI (post-MRI) after the first surgery compared to the pre-operative MRI, which was performed at a relatively large interval prior to resection (8 days before surgery). Retrospectively, an additional tumor volume of 3.1 cm^3^ outside the tumor boundaries segmented in MRI could have been already detected in pre-resectional i3D US but was not interpreted as pathological tissue during surgery. We excluded this case, because, on the one hand, the planned resection was completed, but, on the other hand, remaining tumor could have been seen in i3D US. For more details on tumor object characteristics, refer to Table 2.

**TABLE 2:** Pre-resectional tumor characteristics and iUS quality.

Case	Vol pre-MRI (cm^3^)	Vol pre-US (cm^3^)	Vol res. iUS (cm^3^)	Vol post-US (cm^3^)	Vol post-MRI (cm^3^)	Euclidean Δ CoG (mm)	Hausdorff distance (mm)	Dice coefficient (unitless)	US quality
1	2.4	1.8		0	0	1.11	5.39	0.72	Excellent
2	57.8	57.7	0.2	0	0	4.08	10.20	0.80	Excellent
3	47.9	50.4		17.0	18.1	3.75	15.39	0.80	Excellent
4	5.5	4.84		2.0	1.7	3.35	30.41	0.75	Excellent
5	16.1	15.9		0.8	0.5	1.26	13.34	0.79	Excellent
6	47.8	48.5		0	0	2.06	6.48	0.86	Good
7	18.8	18.4		–	–	3.48	8.06	0.80	Sufficient
8	2.2	2.1		–	–	7.31	9.54	0.36	Good
9	16.4	19.3		0*	0/3.0**	3.19	8.60	0.54	Excellent
10	3.0**	3.1		0	0	7.87	13.89	0.51	Excellent
11	2.4	2.5		0	0	1.23	3.00	0.87	Excellent
12	95.1	90.5		0	0	3.29	8.66	0.86	Good
13	15.7	16.7		0	0	6.07	15.81	0.66	Excellent
14	26.0	24.8		0	0	1.37	9.43	0.77	Sufficient
15	43.9	–		2.1	3.2	–	–	–	Poor
16	22.1	23.1		0.6	0.9	2.40	12.57	0.84	Good
17	6.0	5.6	1.0	0	0	1.21	4.12	0.89	Excellent
18	16.2	16.5		0	0	0.25	19.82	0.92	Excellent
19	30.8	28.7		0	0	3.36	8.06	0.80	Excellent
20	19.7	21.8	3.0	0	0	5.96	31.16	0.64	Sufficient
21	9.2	10.5		0.7	0.1	13.14	14.04	0.29	Sufficient
22	11.0	11.2		2.2	1.7	0.61	4.47	0.91	Good
23	50.8	62.1	2.8	0	0	3.78	11.18	0.79	Good
24	9.6	13.2		2.7	3.3	5.37	14.73	0.69	Sufficient
25	0.5	0.3		0	0	7.64	9.00	0.01	Excellent
26	4.1	4.3		0	0	4.11	13.38	0.50	Excellent
27	51.8	47.1		3.5	2.7	10.63	24.54	0.65	Excellent
28	40.8	35.8		0	0	1.75	13.04	0.86	Good
29	13.0	10.1		0	0	1.96	8.06	0.82	Excellent
30	49.7	38.4		3.7	2.7	3.04	16.79	0.65	Good
31	33.6	34.5		0	0	1.19	7.07	0.93	Excellent

Vol pre-MRI, volume pre-operative segmented in MRI; Vol pre-US, volume pre-resectional segmented in US; Vol res. iUS, volume of residual tumor segmented in i3D US leading to further extension of resection; Vol post-US, volume segmented in last acquired i3D US; Vol post-MR, volume in post-operative MRI; Euclidean Δ CoG, Euclidean difference of geometric center of gravity. Case nos. 9 and 10 are in the same patient within 5 days: * no residual tumor was detected in post-US in case no. 9, but was out of the scanning field and could have been seen in pre-US; ** indicates that no tumor was detected within the pre-operatively segmented area, but a tumor volume of 3.0 cm^3^ was detected in the adjacent in post-MRI (which is also pre-MRI for case no. 10). US quality according to the first author’s subjective assessment.

## Discussion

The dilemma of discordance between pre-operative imaging and intraoperative findings is well known and has been the subject of research in the neurosurgical field for decades. Attempts to overcome this issue reach back to the 1980s, when radiographic imaging of intraoperatively inserted reference objects was used to detect brain movement during surgery (Kelly et al., 1986). Later, landmark studies on brain shift suggested (serial) iMRI for visualization and compensation of deformation during surgical procedures (Nimsky et al., 2000; Nabavi et al., 2001), but in recent years iUS has evolved into a real alternative lacking most of the disadvantages of iMRI (Sastry et al., 2017), such as time consumption, limited availability, high costs, and the need for specialized personnel (Nimsky et al., 2001). In a current retrospective study in 23 patients with different brain tumor entities comparing intraoperative characteristics of iUS and iMRI, Bastos et al. reported a good concordance of tumor localization and margins in iUS with pre-operative MRI and 100% agreement between iUS and iMRI in the prediction of the extent of tumor resection, meaning either STR or GTR as classified by a neuroradiologist in post-MRI (Bastos et al., 2021). This is consistent with our findings of 18 cases classified as GTR and 10 cases as STR in both post-resectional iUS and post-MRI in glioblastoma patients; however, we additionally analyzed the remaining tumor volume in the STR cases, which was 3.5 ± 4.9 cm^3^ (mean ± SD) in i3D US and 3.5 ± 5.2 cm^3^ in post-MRI, showing no significant difference (*p* = 0.8457). This underpins the accuracy of iUS compared to MRI. Further, we identified 4 out of 18 cases (22.2%) in the GTR-group, where i3D US depicted tumor remnants and resection was extended, resulting in no evident remaining tumor in post-resectional iUS or post-MRI.

In light of this and, in contrast, the fact that tumor remnants were reliably identified in all ten STR cases, and in addition, resection was extended in four other cases based on i3D US imaging data, we conclude that although the imaging quality of i3D US is excellent under optimal conditions and may contribute to intraoperative extension of EOR, it cannot replace post-MRI.

Pre-requisite for the evaluation of spatial overlap and potential shifting is a sufficient image fusion and patient registration, which was quantified using the TRE in those 14 cases that received iCT before craniotomy. The TRE of 0.91 ± 0.53 mm is in line with our previously published experience in 200 cases of iCT-based registration in cranial procedures (Carl et al., 2018). The US probe was pre-calibrated, establishing a so-called rigid registration based on spatial position information (Lunn et al., 2003; Prada et al., 2015), and thus, once acquired, i3D US datasets were automatically co-registered to the other imaging modalities. This was analogous to the procedure we described for i3D US in brain metastasis, where we additionally calculated the technical accuracy of US probe pre-calibration to be 1.33 ± 0.33 mm (mean ± SD) utilizing a US phantom containing wires. These were identified in the US image and compared to the expected positions (Saß et al., 2020). In our opinion, reliable registration accuracy can be concluded based on these data, and thus, the presented Dice coefficient C_*DSC*_ of 0.71 ± 0.21 (mean ± SD; median 0.79), which indicates a large spatial overlap, cannot be attributed to the registration procedure to a larger extent. However, multiple factors influence the C_*DSC*_ hindering its approximation toward a value of 1, the so-called perfect match, which one would expect assuming a similar imaging quality in pre-US and pre-MRI. The segmentation procedure itself is error-prone and observer-dependent as shown by Nitsch et al., who had three experts segmenting the same structures in iUS and found a C_*DSC*_ of 0.52–0.83 between the segmentation results and a C_*DSC*_ of 0.74, when an automatic segmentation algorithm was compared to expert segmentation (Nitsch et al., 2019). Another group found wide ranges of C_*DSC*_ reaching from 0.49 to 0.97 for segmentation of brain tumors in MRI when performed by an expert manually or with a semi-automated probabilistic fractional segmentation algorithm (Zou et al., 2004). Similarly, the Multimodal Tumor Image Segmentation Benchmark (BRATS) study, which was designed to compare different automatic segmentation algorithm for brain tumors MRI, analyzed, as a baseline for later comparison with algorithms, the segmentation results of experts and revealed in this regard a considerable disagreement resulting in a C_*DSC*_ of 0.74–0.85 (Menze et al., 2015). Additionally, spatial overlap, and hence the C_*DSC*_, is dependent on the spatial displacement of the segmented object after craniotomy. We chose to determine the spatial shift utilizing the Euclidean distance of the geometric CoG, which resulted in a mean ± SD of 3.9 ± 3.0 mm and a median of 3.3 mm, which is in accordance with our results in brain metastasis surgery, where an Euclidean distance of CoG of 3.7 ± 2.5 mm (mean ± SD, median: 3.0 mm) was seen (Saß et al., 2020). Although it is commonly believed among neurosurgeons that most brain deformation occurs after dural opening, there are several reports claiming a significant shift as early as after craniotomy but before dura incision. Ohue et al. (2010) investigated brain shift by marking reference points in pre-operative imaging and intraoperative US and measuring the distance at different time points. The mean overall shift of all measured structures before dural opening was 2.8 mm, increasing to 4.2 mm immediately before tumor resection, and reached a maximum of 6.8 mm during or after tumor removal (Ohue et al., 2010). Interestingly, when focusing on the measurements made at the tumor margins, which yielded a shift of 3.4 ± 1.9 mm (range: 0.4–10.8 mm) before dural opening, the results of Ohue et al. (2010) are comparable to our measurements. While Ohue et al. (2010) described a significant increase after dura incision, Letteboer et al. (2005) who also measured a shift of 3.0 mm parallel to the direction of gravity, only found additional shifting of 0.2 mm after dura incision. The phenomenon of a higher degree of shifting before than after durotomy can be discussed to be attributed to calibration errors or true shift (Sastry et al., 2017). Because we found the registration error and calibration error of the US probe to be low, we do not expect these factors to affect our measurements to any great extent.

In our experience, mainly three factors can contribute to dissimilarity when comparing pre-operative to intraoperative imaging data, namely, segmentation inaccuracy, pressure applied with a probe during image acquisition, and true brain deformation. While C_*DSC*_ and similar segmentation volumes indicate a good segmentation accuracy, it remains difficult to distinguish between the latter two. To minimize the impact of the probe’s contact with the brain, we used a saline depot as a coupling fluid and applied as little pressure as possible, but still enough to achieve good contact and image quality. However, even the Euclidean distance of the geometric CoG might be to some degree influenced by the probe’s pressure. To further analyze the degree of resemblance of the pre-MRI and pre-US segmentations, we used the Hausdorff distance (Huttenlocher et al., 1993). Here, we found a mean ± SD of 12.3 ± 6.9 mm (and a median of 10.7 mm), which is more than we previously published in case of brain metastasis (mean ± SD: 8.1 mm ± 2.9; median: 8.1 mm) (Saß et al., 2020), but it fits well with the study of Nitsch et al. (2019) who found a Hausdorff distance of 12.2 mm between expert and automated segmentation. To our knowledge, there is no clear definition of when the Hausdorff distance is considered excellent, good, or sufficient. Considering that the Nitsch et al. (2019) results are regarded as “reasonable and acceptable segmentation results,” we interpret our results in the same direction, although the resemblance of tumor objects in pre-MRI and pre-US was higher in brain metastasis (Saß et al., 2020).

Besides our interpretation of tumor volumes, C_*DSC*_, Euclidean distance of CoG, and Hausdorff distance, one must not underestimate the benefit of displaying i3D US datasets as an overlay or site-by-site on the navigational screen, which allows a rough estimation of brain shift and registration coherence. In cases where the registration would become imprecise for any reason, e.g., due to movement of the reference array during the operative course, this could also be detected immediately on the navigational screen. Additionally, in cases where an update of the registration cannot be achieved, it is possible to navigate based on i3D US datasets alone.

The overall tumor identification and delineation was rated by investigators as much more difficult and time consuming than for brain metastases despite the fact that imaging quality was rated to be excellent or good in 25 cases (81%). Keeping this in mind, we interpret the C_*DSC*_, similarity in tumor volume segmentations, and eventually also the Hausdorff distance to indicate a good to excellent segmentation result. We did not use contrast-enhanced ultrasound for our study, which has been shown to improve the capability of distinguishing between tumor and normal brain tissue (Prada et al., 2016). This technique may help refine segmentation results in future studies, but, in contrast, first must be thoroughly integrated into the surgical workflow.

**FIGURE 3:**
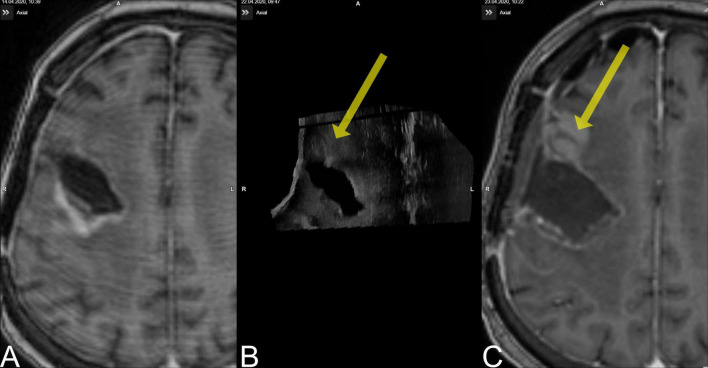
Case no. 9. **(A)** The pre-MRI (8 days prior to surgery) demonstrated a contrast-enhancing lesion occipital to the resection cavity. **(B)** The pre-US demonstrated a lesion rostral to the resection cavity (yellow arrow) that was missed during surgery. **(C)** The new lesion is evident on post-MRI.

A limitation of this study is the exclusion of one case from pre-resectional and three cases from the post-resectional analysis; case no. 15 was a recurrent temporal GBM grade IV, IDH mutant, where we were not able to establish a good contact of the US probe and the scarred dural layer, resulting in exclusion from pre-resectional evaluation; case nos. 7 and 8 were excluded due to corrupted data, case no. 9, which was in the same patient as in case no. 10, is worth a closer scrutiny (Figure 3). The 63–year-old female patient suffered from a recurrent IDH wildtype glioblastoma located at the right frontal lobe. The patient received the pre-MRI 8 days before surgery, and the whole procedure was tailored to the segmented tumor object in pre-MRI. Retrospectively, pre-US during the first surgery documented additional tumor volume (3.1 cm^3^) outside the MRI-segmented object boundaries but was misinterpreted during surgery. The post-US-scanning field, however, did not encompass the additional tumor tissue but was only able to document the MRI-segmented tumor to be completely resected. The tumor mass was then detected in the post-MRI (3.0 cm^3^), and registration to the pre-MRI demonstrated it to be tumor progression. This case is interesting insofar as it demonstrates one of the weaknesses of i3D US; no matter how good the imaging quality of iUS is, the scanning field is limited compared to MRI, and if the examiner focuses only on the pre-operatively segmented areas, pathologies can easily be missed.

## Conclusion

Navigated i3D US in glioblastoma surgery is a helpful tool that allows rough estimation of brain shift and the registration accuracy at a glance even during the surgical procedure, while it can be easily implemented in the surgical workflow. We found a high imaging quality, an excellent concordance of tumor object segmentations compared to post-MRI, and a reliable estimation of residual tumor, which triggered extension of EOR in every fifth case.

## Data Availability Statement

The raw data supporting the conclusions of this article will be made available by the authors, without undue reservation.

## Ethics Statement

The studies involving human participants were reviewed and approved by the Ethics Committee of the Medical Faculty of the Philipps-University Marburg. The patients/participants provided their written informed consent to participate in this study. Written informed consent was obtained from the individual(s) for the publication of any potentially identifiable images or data included in this article.

## Author Contributions

BS, CN, and MB: conceptualization and supervision. MB and BS: methodology and visualization and writing – original draft preparation. DZ, CN, and MP: validation. BS, MB, and DZ: investigation and data curation. MB, DZ, CN, MP, and BS: resources. MB, MP, and CN: writing – review and editing. MB: project administration. All authors have read and agreed to the published version of the manuscript.

## Conflict of Interest

CN and MB are consultants for Brainlab. The remaining authors declare that the research was conducted in the absence of any commercial or financial relationships that could be construed as a potential conflict of interest.

## Publisher’s Note

All claims expressed in this article are solely those of the authors and do not necessarily represent those of their affiliated organizations, or those of the publisher, the editors and the reviewers. Any product that may be evaluated in this article, or claim that may be made by its manufacturer, is not guaranteed or endorsed by the publisher.
